# Diurnal pattern of breaks in sedentary time and the physical function of older adults

**DOI:** 10.1186/s13690-023-01050-1

**Published:** 2023-03-06

**Authors:** Ting-Fu Lai, Yung Liao, Chien-Yu Lin, Ming-Chun Hsueh, Mohammad Javad Koohsari, Ai Shibata, Koichiro Oka, Ding-Cheng Chan

**Affiliations:** 1 Department of Health Promotion and Health Education, National Taiwan Normal University, Taipei City, Taiwan; 2 Graduate Institute of Sport, Leisure and Hospitality Management, National Taiwan Normal University, Taipei City, Taiwan; 3 Faculty of Sport Sciences, Waseda University, Tokorozawa, Japan; 4 Graduate Institute of Sport Pedagogy, University of Taipei, Taipei City, Taiwan; 5 School of Knowledge Science, Japan Advanced Institute of Science and Technology, Ishikawa, Japan; 6 Faculty of Health and Sport Sciences, University of Tsukuba, Tsukuba, Japan; 7 Department of Geriatrics and Gerontology, National Taiwan University Hospital, Taipei City, 100 Taiwan; 8 Department of Internal Medicine, National Taiwan University Hospital, Taipei City, Taiwan; 9 Master’s Program of Transition and Leisure Education for Individuals with Disabilities, University of Taipei, Taipei, Taiwan

**Keywords:** Aging, Interrupting prolonged sitting, Circadian clock, Elderly

## Abstract

**Background:**

The association of breaks in sedentary time with outcomes of physical function can vary according to the time of day. We examined the association of the diurnal pattern of breaks in sedentary time with physical function outcomes in older adults.

**Methods:**

A cross-sectional analysis was conducted among 115 older adults (≥60 years). The overall and time-specific breaks (morning: 06:00–12:00; afternoon: 12:00–18:00; evening: 18:00–24:00) in sedentary time were assessed using a triaxial accelerometer (Actigraph GT3X+). A break in sedentary time was defined as at least 1 min where the accelerometer registered ≥100 cpm following a sedentary period. Five physical function outcomes were assessed: handgrip strength (dynamometer), balance ability (single leg stance), gait speed (11-m walking), basic functional mobility (time up and go), and lower-limb strength (five times sit-to-stand). Generalized linear models were used to examine the associations of the overall and time-specific breaks in sedentary time with the physical function outcomes.

**Results:**

Participants showed an average of 69.4 breaks in sedentary time during the day. Less frequent breaks in the evening (19.3) were found than that in the morning (24.3) and the afternoon (25.3) (*p* < 0.05). Breaks in sedentary time during the day were associated with less time on gait speed in older adults (exp (β) = 0.92, 95% confidence interval [CI] 0.86–0.98; *p* < 0.01). Time-specific analysis showed that breaks in sedentary time were associated with less time on gait speed (exp (β) = 0.94, 95% CI 0.91–0.97; *p* < 0.01), basic functional mobility (exp (β) = 0.93, 95% CI 0.89–0.97; *p* < 0.01), and lower-limb strength (exp (β) = 0.92, 95% CI 0.87–0.97; *p* < 0.01) in the evening only.

**Conclusion:**

A break in sedentary time, particularly during the evening, was associated with better lower extremity strength in older adults. Further strategies to interrupt sedentary time with frequent breaks, with an emphasis on evening hours, can be helpful to maintain and improve physical function in older adults.

## Background

The number and proportion of the individuals 65 years and older are growing worldwide—the number is expected to more than double from 2019 to 2050, and the proportion is projected to rise from 9% in 2019 to 16% in 2050 [1]. Aging is associated with some decline in health status [2]. A decline in physical function is considered the main reason for a decline in physical independence and an increased risk of disability [3]. Globally, it is estimated that more than 46% of adults over 60 years of age have disabilities and at least 250 million had moderate to severe disability in 2012 [4]. The impact of disability on older adults places a heavy burden of medical expenses [5] and mental stress on family caregivers [6]. Identifying modifiable correlates of physical function may be promising in preventing disability in older adults and reducing the socioeconomic burden.

An updated global guideline on physical activity and sedentary behavior suggests that reduced sedentary time is independent of preventing a decline in physical function in older people [7]. Breaking up prolonged sedentary time can provide opportunities to intervene rather than increasing physical activity. Targeting the older population would be a priority, as some evidence shows this age group may spend most of their waking hours in sedentary behavior [8]. A recent meta-analysis suggests that increased breaks in sedentary time are associated with increased muscle strength in older adults [9]. However, there are limited studies investigating the diurnal pattern in older adults of the association between breaks in sedentary time and physical function [10]. The time spent on physical activity can be restricted in the older population more so than in younger groups due to physiological deterioration. Therefore, investigations of diurnal break patterns in sedentary time and physical function could contribute to the information on the recommended time of day for the projected additional benefits, while designing effective interventions or strategies to maintain and improve muscle function in older adults [11].

This study investigated the associations between breaks in sedentary time, during the day and at different times of the day, and physical function outcomes among older adults. It has been shown that older adults with higher levels of energy intake (e.g., carbohydrate intake with glucose fluctuations) in the evening are associated with lower muscle mass than that in the morning and afternoon [12, 13]. Therefore, it is important to examine whether frequent breaks in sedentary time, with more energy expenditure, during the evening have the most marked association with physical function in older adults than that at other times of the day.

## Methods

### Participants

We recruited older adults in the community aged 60 years and over, using local advertisements and voice announcements at the community centers of 28 selected neighborhoods in Taipei City, Taiwan. Those who were unable to walk independently (i.e., a need to use walking assistance equipment or help with someone’s arm) were excluded from this study. Detailed recruitment procedures have been reported in a previous study [14]. The minimum sample size required was determined using effect size of d = 0.25, with power of 0.8, and the alpha level was set to 0.05 using the G*Power software. To acquire the sample size that should be recruited in the beginning, the minimum sample size calculated was then back-calculated after considering the attrition rate of 10%. The number of participants recruited in the beginning was at least 111. A total of 130 older adults participated in the study; 115 had on-site examinations and wore a three-axis accelerometer for seven consecutive days with sufficient valid days. A valid day was defined as wearing an actigraphy device for 10 h or more during waking hours. Data with at least 4 valid days (including 1 weekend day) were included in the analysis. This approach was consistent with previous studies [14, 15]. Informed consent was obtained from each participant before participation in the study. The Research Ethics Committee of the National Taiwan Normal University (REC number: 201706HM020) approved the study.

### Outcomes

Physical function outcomes, including upper extremity strength (i.e., handgrip strength), balance ability, and lower extremity strength (i.e., gait speed, basic functional mobility, and lower-limb strength), were assessed by five independent on-site examinations [16, 17]. The handgrip strength of both hands was measured in turn using the hydraulic hand dynamometer (Jamar Plus+ Digital Hand Dynamometer 5632–13). We asked the participants to sit with their back supported, hips and knees flexed at 90°, feet in contact with the ground, elbows flexed at 90°, with forearms and wrists in neutral position. The participants were then asked to hold the dynamometer and squeeze with as much force as possible. The optimum performance of higher strength was selected from two attempts with a 1 min break in between. We measured balance ability using single leg stance test. The participants were asked to stand on one leg with shoes and their eyes open, lift one leg off the floor, and the time taken until they lowered their foot down to the floor was recorded [18]. The maximum time was recorded as 60 s if the time was prolonged more than 60 s. To measure gait speed, each participant was asked to walk 11 m in one direction as fast as possible [19]. The shorter walk time in the central 5 m was estimated as the optimum gait speed. To assess basic functional mobility, we also asked participants to rise from a conventional chair (seat height: 43 cm), walk 3 m forward, turn, return to the chair, and sit down as quickly as possible [20]. To assess their lower-limb strength, participants were asked to sit on a conventional chair (same as basic functional mobility) and repeat stand up and sit down 5 times, as fast as possible [21]. Each participant repeated the same procedure twice for basic functional mobility and lower-limb strength. The optimum performance selected was the shortest time for each measurement.

### Exposures

Time spent on sedentary behaviors (≤ 99 cpm) was measured by a triaxial accelerometer (ActiGraph GT3X+) worn on the waist [22]. A break in a sedentary time was defined as at least 1 min when the accelerometer registered ≥100 cpm following a sedentary period, according to previous definitions [23, 24]. We followed the data collection and processing criteria procedure suggested by a systematic review of standard protocols for the use of accelerometers [25]. Non-wear time was identified while the periods of more than 60 consecutive minutes of zero counts. The participants were also asked to assess their sleep time and duration using a sleep log. Time intervals during the waking hours were identified, in which sedentary breaks occurred in the morning (06:00–12:00), afternoon (12:00–18:00) and evening (18:00–24:00) based on previous studies [26, 27]. ActiLife software version 6.0 was used to extract the accelerometer data; all data were processed using 60-s time-spans with a default sampling frequency of 30 Hz.

### Covariates

Interviewer-administered questionnaires were used to collect the age of the participants (60–74 or ≥ 75 years), sex, marital status (married or not married), living status (living alone or with others), educational level (having a university degree or lower), health status and habitual behaviors. The height and weight of the participants was measured by trained personnel. Health status included general health measured by a five-point Likert scale (1-very bad to 5-very good in response to, “In general, do you consider yourself to be healthy?”), mental health (a yes/no response to “Did you frequently feel depressed in the past month?”), and physical health. Responses to general health questions with at least three points were classified as “good,” and others as “bad.” Physical health identified chronic disease, such as diabetes, hypertension, and hyperlipidemia. Body mass index (BMI) was calculated using height and weight and classified into “normal (18–24 kg/m^2^)” or “overweight (> 24 kg/m^2^)” [28]. Habitual behaviors were alcohol consumption, tobacco use, and a balanced diet according to the Taiwan national standard [28]. The time measured by the accelerometer in moderate to vigorous physical activity (MVPA), identified as ≥2020 cpm [22], was classified as “sufficient (≥ 150 min/week)” and “insufficient (< 150 min/week)” [7]. The total sedentary time and wear-time of the accelerometer were also included.

### Statistical analysis

We used descriptive statistics to show the mean and standard deviation (SD) of the physical function outcomes. We selected covariates for the different outcomes of physical function using independent sample t-tests. The relevant covariates for each physical function outcome were identified when there were statistical differences in the characteristics of the participants and were adjusted in the corresponding models (see Table 1. Descriptive statistics between the characteristics of the participants and outcomes of physical function (*n* = 115)). All generalized linear models were further controlled for overall MVPA, sedentary time, and monitor wear-time. Repeated analysis of variance (ANOVA) was used to determine the difference in breaks in sedentary time across the three different times of the day, due to dependent events. The bar charts with the mean and SD of the overall breaks and time-specific breaks in sedentary time were presented. Generalized linear models specifying a gamma distribution with a log link were used to examine the associations of breaks in sedentary time with physical function outcomes. The antilogarithms of the regression coefficients (exp[β]) and 95% confidence intervals (CIs) were estimated after controlling for appropriate covariates. Regression coefficients represented proportional changes in strength or time spent performing physical function. All analyses were performed using IBM SPSS Statistics 23.0. The significance level was established at *p* < 0.05.

**Table 1:** Descriptive statistics between the characteristics of the participants and outcomes of physical function (*n* = 115)

Characteristics	Total N (%)	Outcome of physical function				
		Handgrip strength (kg), mean (SD)	Balance ability (sec), mean (SD)	Gait speed (sec), mean (SD)	Basic functional ability (sec), mean (SD)	Lower- limb strength (sec), mean (SD)
Sex		*p < 0.01	*p* = 0.35	*p = 0.01	*p* = 0.31	*p* = 0.79
Men	31 (27.0)	33.2 (6.0)	39.9 (23.1)	2.8 (0.5)	6.8 (1.8)	7.6 (2.1)
Women	84 (73.0)	21.5 (3.5)	35.3 (23.1)	3.1 (0.7)	7.2 (1.8)	7.4 (2.7)
Age group (year)		*p* = 0.20	*p < 0.01	*p < 0.01	*p < 0.01	*p = 0.03
60–74	94 (81.7)	25.1 (6.7)	40.8 (22.2)	2.9 (0.6)	6.7 (1.6)	7.2 (2.6)
≥ 75	21 (18.3)	22.9 (6.7)	17.5 (16.9)	3.6 (0.7)	8.6 (1.9)	8.5 (2.2)
BMI		*p* = 0.50	*p* = 0.05	*p < 0.01	*p < 0.01	*p* = 0.15
Normal	59 (51.3)	24.3 (6.1)	40.6 (21.5)	2.8 (0.6)	6.6 (1.4)	7.1 (2.4)
Overweight	56 (48.7)	25.1 (7.4)	32.2 (24.0)	3.2 (0.7)	7.5 (2.0)	7.8 (2.7)
Marital status		*p < 0.01	*p = 0.01	*p = 0.04	*p* = 0.17	*p* = 0.96
Married	77 (67.0)	25.9 (7.5)	40.3 (22.5)	2.9 (0.6)	6.9 (1.8)	7.4 (2.7)
Not married	38 (33.0)	22.3 (4.2)	28.9 (22.5)	3.2 (0.7)	7.4 (1.7)	7.5 (2.3)
Living status		*p* = 0.10	*p* = 0.53	*p* = 0.43	*p* = 0.27	*p = 0.03
Living alone	12 (10.4)	22.8 (3.5)	32.6 (22.6)	3.1 (0.8)	7.6 (2.1)	9.0 (3.5)
Living with others	103 (89.6)	24.9 (7.0)	37.0 (23.2)	3.0 (0.6)	7.0 (1.7)	7.3 (2.4)
Educational level		*p* = 0.33	*p < 0.01	*p < 0.01	*p* = 0.05	*p* = 0.59
University degree	26 (22.6)	25.8 (8.1)	49.7 (18.1)	2.6 (0.5)	6.2 (1.2)	7.2 (2.9)
Lower than university degree	89 (77.4)	24.4 (6.3)	32.7 (23.0)	3.1 (0.7)	7.3 (1.8)	7.5 (2.5)
Employment		*p* = 0.10	*p* = 0.53	*p* = 0.63	*p* = 0.26	*p* = 0.43
With a full-time job	4 (3.5)	19.2 (1.7)	29.3 (28.9)	3.2 (0.7)	8.0 (2.1)	8.5 (3.7)
Without a full-time job	111 (96.5)	24.9 (6.8)	36.8 (23.0)	3.0 (0.7)	7.0 (1.8)	7.4 (2.5)
General health		*p = 0.02	*p < 0.01	*p < 0.01	*p < 0.01	*p = 0.01
Good	36 (31.3)	27.1 (7.6)	44.8 (20.8)	2.7 (0.6)	6.4 (1.6)	6.6 (1.9)
Bad	79 (68.7)	23.6 (6.1)	32.8 (23.2)	3.1 (0.6)	7.4 (1.8)	7.9 (2.7)
Frequent depressed		*p < 0.01	*p = 0.01	*p* = 0.14	*p* = 0.08	*p* = 0.27
Yes	15 (13.0)	21.2 (4.3)	22.3 (20.8)	3.3 (0.7)	7.8 (2.2)	8.4 (3.7)
No	100 (87.0)	25.2 (6.9)	38.7 (22.7)	3.0 (0.6)	6.9 (1.7)	7.3 (2.3)
Diabetes		*p* = 0.53	*p = 0.03	*p* = 0.47	*p* = 0.18	*p* = 0.29
Yes	19 (16.5)	23.8 (6.1)	26.3 (22.6)	3.1 (0.7)	7.6 (2.1)	8.0 (3.5)
No	96 (83.5)	24.9 (6.9)	38.5 (22.7)	3.0 (0.7)	7.0 (1.7)	7.3 (2.4)
Hypertension		*p* = 0.50	*p* = 0.28	*p* = 0.40	*p* = 0.25	*p* = 0.33
Yes	45 (39.1)	25.2 (6.9)	33.6 (23.9)	3.1 (0.7)	7.3 (1.7)	7.7 (2.6)
No	70 (60.9)	24.3 (6.7)	38.4 (22.5)	3.0 (0.7)	6.9 (1.8)	7.3 (2.5)
Hyperlipidemia		*p* = 0.99	*p* = 0.28	*p* = 0.14	*p* = 0.06	*p* = 0.42
Yes	33 (28.7)	24.7 (6.8)	32.8 (23.7)	3.1 (0.7)	7.6 (1.9)	7.8 (2.5)
No	82 (71.3)	24.7 (6.8)	38.0 (22.8)	2.9 (0.6)	6.9 (1.7)	7.3 (2.6)
Alcohol use		*p* = 0.50	*p = 0.03	*p* = 0.73	*p* = 0.62	*p* = 0.93
Yes	9 (7.8)	26.2 (7.2)	20.7 (21.1)	3.1 (0.7)	7.3 (2.3)	7.4 (1.8)
No	106 (92.2)	24.6 (6.7)	37.9 (22.8)	3.0 (0.7)	1.7 (1.7)	7.5 (2.6)
Cigarette use		*p < 0.01	*p* = 0.81	*p* = 0.17	*p* = 0.29	*p* = 0.27
Yes	7 (6.1)	31.5 (8.3)	38.6 (27.0)	2.7 (0.4)	6.4 (1.1)	6.4 (1.5)
No	108 (93.9)	24.2 (6.4)	36.4 (22.9)	3.0 (0.7)	7.1 (1.8)	7.5 (2.6)
Dietary intake		*p < 0.01	*p* = 0.34	*p = 0.03	*p* = 0.18	*p* = 0.79
Balanced	85 (73.9)	25.6 (7.1)	37.8 (23.1)	2.9 (0.6)	6.9 (1.7)	7.4 (2.7)
Unbalanced	30 (26.1)	22.1 (4.7)	33.1 (22.9)	3.2 (0.8)	7.4 (2.0)	7.6 (2.3)
MVPA		*p < 0.01	*p < 0.01	*p < 0.01	*p < 0.01	*p < 0.01
Insufficient	59 (51.3)	23.1 (6.5)	30.3 (24.0)	3.3 (0.7)	7.7 (2.0)	8.1 (3.1)
Sufficient	56 (48.7)	26.4 (6.7)	43.1 (20.2)	2.7 (0.5)	6.4 (1.2)	6.8 (1.7)

*BMI* body mass index, *SD* standard deviation, *MVPA* Moderate-to-vigorous physical activity

*p < 0.05

## Results

**Fig. 1:**
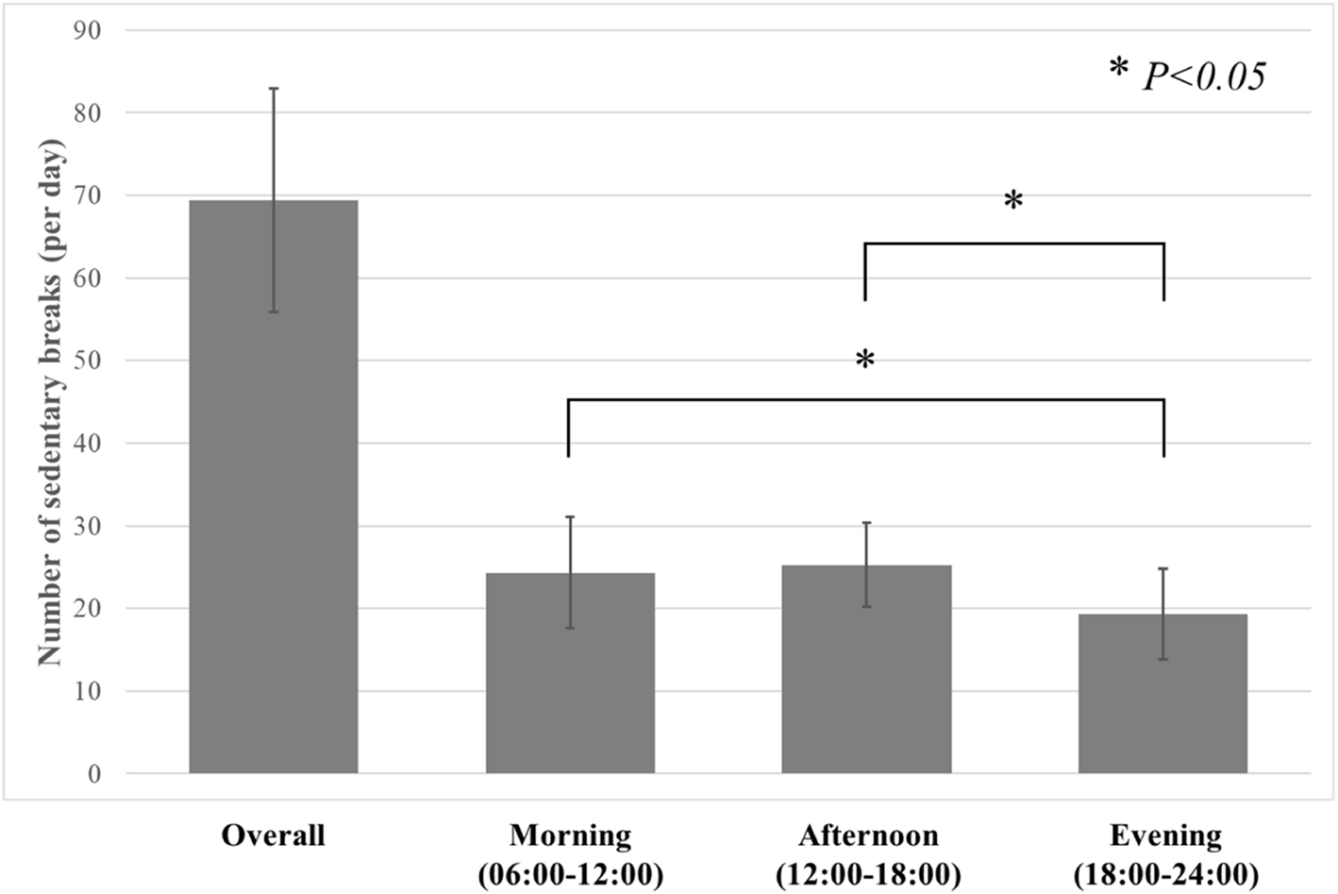
One way ANOVA test was applied for Fig. 1. Data were expressed as mean ± SD

Almost three-quarters of the participants were women (73.0%), and more than four-fifths were aged 60–74 years (81.7%). Table 1 shows that there were some differences in physical function performance between the characteristics of the participants. For example, those who met the recommended level of physical activity showed higher handgrip strength (26.4 kg vs 23.1 kg), remained balanced for longer (43.1 s vs 30.3 s), walked faster for the same distance (2.7 s vs 3.3 s), moved faster for basic functional mobility (6.4 s vs 7.7 s), and changed their posture faster in the sit-stand test (6.8 s vs 8.1 s). The time spent using the accelerometer was 15.4 h and the total sedentary time during a day averaged 10.1 h. Sedentary time was distributed equally in the morning (2.9 h), afternoon (3.6 h), and in the evening (3.1 h). Figure 1 shows an average of 69.4 (SD = 13.5) breaks in sedentary time during the day. The breaks in sedentary time were similar in the morning (24.3 ± 6.7) and the afternoon (25.3 ± 5.1), but less frequent in the evening (19.3 ± 5.5) (*p* < 0.05).

An increase in one SD of breaks in sedentary time during the day was associated with a shorter time on the gait speed test (exp (β) = 0.92, 95% CI 0.86–0.98; *p* < 0.01), but not associated with other physical function outcomes (Table 2). Time-specific analysis showed that an increase in SD of breaks in sedentary time during the evening was associated with a shorter time on the gait speed test (exp (β) = 0.94, 95% CI 0.91–0.97), basic functional mobility (exp (β) = 0.93, 95% CI 0.89–0.97), and lower-limb strength (exp (β) = 0.92, 95% CI 0.87–0.97). There were no associations between the study variables in the morning or afternoon.

**Table 2:** Associations of the overall and time-specific breaks in sedentary time with the outcomes of physical function (*n* = 115)

Outcome of physical function	Overall breaks in sedentary time during the day (z-score)			Time-specific breaks in sedentary time during the day (z-score)								
				Morning		Afternoon			Evening			
				(06:00–12:00)		(12:00–18:00)			(18:00–24:00)			
	Exp (β)	(95% CI)	p	Exp (β)	(95% CI)	p	Exp (β)	(95% CI)	p	Exp (β)	(95% CI)	p
^1^Handgrip strength (kg)^a^	0.98	(0.92, 1.05)	0.61	1.00	(0.95, 1.05)	0.99	1.01	(0.97, 1.05)	0.67	0.99	(0.95, 1.02)	0.44
^2^Balance ability (s)^a^	1.16	(0.88, 1.52)	0.29	1.18	(0.95, 1.47)	0.14	0.97	(0.81, 1.16)	0.69	1.06	(0.89, 1.27)	0.50
^3^Gait speed (s) ^b^	**0.92**	**(0.86, 0.96)**	**< 0.01**	0.97	(0.93, 1.02)	0.24	0.99	(0.96, 1.03)	0.81	**0.94**	**(0.91, 0.97)**	**< 0.01**
^4^Basic functional mobility (s)^b^	0.95	(0.89, 1.02)	0.14	1.00	(0.95, 1.05)	0.90	0.99	(0.94, 1.03)	0.58	**0.93**	**(0.89, 0.97)**	**< 0.01**
^5^Lower-limb strength (s)^b^	0.91	(0.83, 1.00)	0.056	0.98	(0.91, 1.05)	0.56	1.00	(0.93, 1.07)	0.91	**0.92**	**(0.87, 0.97)**	**< 0.01**

exp (β): antilogarithms of the regression coefficients; CI: confidence interval. The estimates with *p* < 0.05 was highlighted in bold

^1^ Adjusted for sex, marital status, self-rated health, depression, tobacco use, dietary intake, MVPA, sedentary time, and monitor wear time

^2^ Adjusted for age group, marital status, education level, self-rated health, depression, diabetes, alcohol use, MVPA, sedentary time, and monitor wear time

^3^ Adjusted for sex, age group, BMI, marital status, educational level, self-rated health, dietary intake, MVPA, sedentary time, and monitor wear time

^4^ Adjusted for age group, BMI, self-rated health, MVPA, sedentary time, and monitor wear time

^5^ Adjusted for age group, living status, MVPA, sedentary time, and monitor wear time

^a^ A positive association indicates a better physical function, accompanied by a higher break in sedentary time

^b^ A negative association indicates a better physical function, accompanied by a higher break in sedentary time

## Discussion

This is the first study, to our knowledge, to examine the associations of the diurnal pattern of breaks in sedentary time with the outcomes of physical function in older adults. The overall breaks in sedentary time throughout the day was associated with better gait speed test and breaks in sedentary time in the evening were associated with better lower extremity strength, indicated by outcomes of gait speed, basic functional mobility, and lower-limb strength.

In keeping with the present study findings, a previous study also showed that frequent breaks in sedentary time were associated with improved lower extremity function [29]. It has been suggested that breaking up sedentary time with frequent active breaks throughout the day can lead to increased skeletal muscle strength due to increased opportunities for muscular contractions [9]. However, in contrast to some previous studies [30, 31], we found no association with the overall breaks in sedentary time during the day with basic function mobility or lower-limb strength. A possible explanation is that a stronger stimulus than a muscular contraction originates from a break in sedentary time may be needed to induce an improvement in physical function outcomes, such as handgrip strength and lower limb strength [32]. Furthermore, a previous study also indicated that a break in sedentary time was not related to subjective physical function, taking into account MVPA and the duration of the sedentary period [33]. Another possible explanation for the non-significant associations found in our study could be that the participants’ characteristics [30, 31]. The mean age of the participants in the present study was 70 years, while those in previous studies were 73 and 75 years, respectively [30, 31]. An average time of 7.7 s for functional mobility was shown by Sardinha et al. [31] and an average time of 11.3 s for lower limb strength was shown by Wilson et al. [30]. The younger participants in this study generally performed better physical funtion outcomes with spending shorter average time on basic functional mobility (7.1 s) and lower limb strength (7.46 s) than that in previous studies using the same measures [30, 31].

Regarding different times of day, the better outcomes of lower extremity strength, including gait speed, basic functional mobility, and lower limb strength, were associated with a frequent break in sedentary time in the evening, but not in the morning or afternoon. Although no similar studies have investigated the association between breaks in sedentary time and physical function outcomes across times of the day, a recent study suggested that the breaks in sedentary time in the evening showed positive associations with a higher percentage of optimum glycemic indices, in patients with diabetes, after dinner and at bedtime [34]. Glucose fluctuations, independently associated with sarcopenia, low muscle mass, less grip strength, and slow gait speed, in patients with diabetes [13], could be related to physical function. Generally, people consume more carbohydrates at dinner leading to higher glucose fluctuations than with the other two meals of the day [12], bearing in mind it is crucial to stabilize blood glucose levels of patients with diabetes in the evening. Older people who take more breaks from sedentary time in the evening are more likely to have better glucose management and thus contribute to optimum physical function outcomes. Future research is needed to investigate the underlying pathways between breaks in sedentary time, glycemic fluctuations, and physical function outcomes among older adults. Furthermore, a previous study indicated that older adults reached the highest level of physical activity during the day to perform daily tasks such as running errands and voluntary exercise [35]. Higher levels of physical activity during the day can be accompanied by frequent breaks in sedentary time in the morning and afternoon than in the evening. The smaller variations in the breaks in sedentary time may attenuate the relationships in question during the day.

One strength of this study was to use objective measures to assess both the breaks in sedentary time and the outcomes of physical function. There are some limitations to consider when interpreting the results. First, the sample size was relatively small, and most of the participants were women; the participants may not be representative of all older adults in Taiwan. Future research with a large number of participants and representative sample that investigates the relationships is needed. Second, the three different times during the day were identified based on the assumption of waking hours when people can change behavior (e.g., from sitting to standing). There may be inconsistencies in the onset and duration of sleep of participants. However, our data showed that almost 90% of the participants slept and covered the period, 00:00–06:00, without any sedentary or physically active behavior. Third, the accelerometer data for identifying breaks in sedentary time used a sampling frequency (i.e., 30 Hz) following previous research. However, the setting for sampling frequency may be too wide to identify changes in the position, particularly among older adults. Future studies using accelerometer data with a setting for lower frequency are needed. Fourth, there may be some covariates not assessed. For example, a previous study from Portugal has shown that older women performed more frequent breaks in sedentary time were less likely to present unfavorable waist circumference but not associated with BMI [36]. In this study, we measured BMI and considered it as the covariates in the regression models when applicable. Further unmeasured covariates and indexes should be considered. Finally, the cross-sectional association between the breaks in sedentary time and the outcomes of physical function observed in this study could not imply causality.

## Conclusions

The study found that older adults who had more frequent breaks in sedentary time during the evening had better outcomes of lower extremity strength, including gait speed, basic functional mobility, and lower-limb strength. The findings suggest that increases in evening breaks while sedentary may be beneficial to the strength of the lower extremities in older adults. Strategies or interventions relevant to interrupting sedentary time with frequent breaks in the evening must be developed to maintain and improve the physical functional ability of older adults.

## Data Availability

Data used in this study are available upon reasonable request.

## Abbreviations

SD: Standard deviation
CIs: Confidence intervals
